# Osteoprotegerin is a marker of cardiovascular mortality in patients with chronic kidney disease stages 3–5

**DOI:** 10.1038/s41598-021-82072-z

**Published:** 2021-01-28

**Authors:** Gustavo Lenci Marques, Shirley Hayashi, Anna Bjällmark, Matilda Larsson, Miguel Riella, Marcia Olandoski, Bengt Lindholm, Marcelo Mazza Nascimento

**Affiliations:** 1 Department of Internal Medicine, Hospital de Clínicas, Federal University of Paraná, General Carneiro, 181, Curitiba, PR 80060-900 Brazil; 2 Divisions of Renal Medicine and Baxter Novum, Department of Clinical Science, Intervention and Technology, Karolinska Institute, Karolinska University Hospital in Huddinge, Stockholm, Sweden; 3 School of Health and Welfare, Jönköping University, Jönköping, Sweden; 4 Royal Institute of Technology, Stockholm, Sweden; 5 ProRenal Foundation, Curitiba, PR Brazil; 6 Pontifícia Universidade Católica Do Paraná, Curitiba, PR Brazil

**Keywords:** Chronic kidney disease, Calcification, Cardiovascular diseases

## Abstract

Cardiovascular disease (CVD) is the leading cause of death in patients with chronic kidney disease (CKD). Osteoprotegerin (OPG), known to regulate bone mass by inhibiting osteoclast differentiation and activation, might also play a role in vascular calcification. Increased circulating OPG levels in patients with CKD are associated with aortic calcification and increased mortality. We assessed the predictive role of OPG for all-cause and cardiovascular mortality in patients with CKD stages 3–5 over a 5-year follow-up period. We evaluated the relationship between OPG and all-cause and cardiovascular mortality in 145 CKD patients (stages 3–5) in a prospective observational follow-up study. Inflammation markers, including high-sensitivity C-reactive protein, standard echocardiography, and estimation of intima-media thickness in the common carotid artery, were assessed at baseline, and correlations with OPG levels were determined. The cutoff values for OPG were defined using ROC curves for cardiovascular mortality. Survival was assessed during follow up lasting for up to 5.5 years using Fine and Gray model. A total of 145 (89 men; age 58.9 ± 15.0 years) were followed up. The cutoff value for OPG determined using ROC was 10 pmol/L for general causes mortality and 10.08 pmol/L for CV causes mortality. Patients with higher serum OPG levels presented with higher mortality rates compared to patients with lower levels. Aalen–Johansen cumulative incidence curve analysis demonstrated significantly worse survival rates in individuals with higher baseline OPG levels for all-cause and cardiovascular mortality (*p* < 0.001). In multivariate analysis, OPG was a marker of general and cardiovascular mortality independent of sex, age, CVD, diabetes, and CRP levels. When CKD stages were included in the multivariate analysis, OPG was an independent marker of all-cause mortality but not cardiovascular mortality. Elevated serum OPG levels were associated with higher all-cause and cardiovascular mortality risk, independent of age, CVD, diabetes, and inflammatory markers, in patients with CKD.

## Introduction

Cardiovascular disease (CVD) accounts for about 30% of deaths worldwide^1^ and is the leading cause of death in patients with chronic kidney disease (CKD)^2^. However, the high prevalence of traditional risk factors cannot fully explain the high incidence of fatal events in this population^3, 4^. Therefore, in the last decade, other risk factors, such as markers of inflammation, oxidative stress, and vascular calcification, have been studied^5–7^. In this context, osteoprotegerin (OPG) has become the subject of increased interest because of its role as a cardiovascular risk factor in both the general population as well as in CKD patients^8^.

OPG is a soluble receptor of the osteoclast activator, receptor activator of nuclear factor-κB ligand (RANKL), and has been studied as an important marker of CVD in the pathogenesis of vascular calcification and atherosclerosis^9, 10^. Elevated serum OPG levels seem to be associated with increased morbidity and mortality in patients with coronary artery disease and heart failure. A recent meta-analysis demonstrated a significant association between circulating OPG levels and mortality in the general population^11^.

OPG has also been associated with higher mortality in patients undergoing hemodialysis^12^. In a preliminary observational study, our research group found a significant association between elevated OPG levels and the presence of increased all-cause mortality in a cohort of patients with CKD stages 3–5 over a 3-year follow-up period^13^. To date, our research group has not studied the association between elevated serum OPG levels and cardiovascular mortality or its interaction with other myocardial injury markers.

We hypothesized a possible correlation between elevated serum OPG levels in patients with CKD stages 3–5 and markers of presence and severity of CVD, and investigated whether OPG is a possible independent marker of cardiovascular mortality in patients with CKD over a 5-year follow-up period.

## Materials and methods

Patients with CKD stages 3–5, including those undergoing dialysis at Pró-Renal Foundation in Curitiba, Brazil, were considered for enrollment in this study. All patients gave written informed consent and the ethics committee of the Hospital Evangelico de Curitiba approved the study protocol and all the study procedures were carried out in accordance with relevant guidelines. The exclusion criteria were dialysis treatment lasting for less than 1 month, age younger than 18 years, the presence of HIV or hepatitis B/C infection, and other chronic inflammatory diseases.

### Study design

All patients enrolled through the cohort study design underwent a baseline investigation comprising blood sampling and cardiovascular assessment. They were subsequently followed up for up to 66 months for the analysis of survival. The observation period was from March 2008 to December 2015.

### Cardiovascular and laboratory assessment

The intima-media thickness (IMT) was evaluated using semiautomatic edge detection software (GE Vingmed Ultrasound, Horten, Norway) according to the recommendations of the American Society of Echocardiography^14^. All two-dimensional and Doppler variables were acquired and analyzed according to the guidelines of the American Society of Echocardiography^15, 16^. All ultrasound examinations were performed only once, when the patient was included in the cohort.

Blood samples were collected from patients after overnight fasting. The samples were collected midweek from hemodialysis patients and at regular clinic visits from other patients with CKD stages 3–5, including those undergoing peritoneal dialysis. All analyses were performed using automated analyzers at the Renal Medicine Laboratory, Clinical Research Center, Karolinska Institutet, Stockholm, Sweden^13^. Serum OPG was measured using a ELISA assay commercial kit (R&D Systems Inc.). Plasma and serum were stored at − 70 °C for transport, the analyzes were carried out as soon as the material reached its destination.

### Statistical analysis

Data are reported as median (interquartile range) or mean ± standard deviation (SD), and as frequencies and percentages for categorical variables, as appropriate. For determining cutoff values for OPG associated with cardiovascular death, receiver operating characteristic (ROC) curves were adjusted and the corresponding area under curve (AUC) was evaluated. Best cutoff was determined using Youden index criteria. To compare two groups with continuous quantitative variables, we used a Student’s t-test for independent samples. The non-normally distributed variables were log-transformed and parametric tests were applied. We used Fisher's exact test for categorical variables. Three groups were compared using Chi-square test or one-way analysis of variance (ANOVA) and Bonferroni post-hoc test. For analyzing factors associated with the OPG (pg/mL) level initially, a univariate analysis was performed estimating Spearman's correlation coefficients. Subsequently, a multivariate linear regression model was adjusted, including as explanatory variables those that presented significant correlations with OPG in the univariate analysis. The response variable for multiple linear regression was log-OPG and included log-transformed explanatory variables. Residuals were checked by normal probability plot and predicted values versus residuals scatterplot. Fine and Gray models were adjusted to analyze mortality. For all-cause mortality models, transplantation was considered as the competing risk. For cardiovascular models, other causes of mortality and transplantation were considered as competing risks. The estimated association measure was the subdistribution hazard ratio (SHR) provided for unit change with 95% confidence interval. Aalen–Johansen cumulative incidence curves were presented for OPG. The selection of variables for multivariate analysis models used clinically relevant predictors. The first multivariate analysis model that used OPG added age and gender. The second model used the same variables as the first one and included the presence of established CVD and diabetes. The third model used all previous variables and included an inflammatory activity marker, C-reactive protein (hsCRP). The final model included all the aforementioned variables, and patients CKD stages. The normality of the variables was evaluated using the Kolmogorov–Smirnov test. *p* values < 0.05 were considered statistically significant. Data were analyzed using Stata/SE v. 14.1 (StataCorp LP, College Station, TX, USA).

## Results

### Baseline characteristics

Altogether, 145 (89 males) patients, including 54 non-dialysis patients with CKD stages 3–5, 36 hemodialysis (HD) patients, and 55 peritoneal dialysis patients, fulfilled the study eligibility criteria and agreed to participate in the study. All dialysis patients did not present residual diuresis. Of the patients under conservative treatment, 9 started dialysis therapy during the period observed. The median follow-up was 36, 5 months, the minimum 0, 4 month and the maximum 66 months.

**Figure 1:**
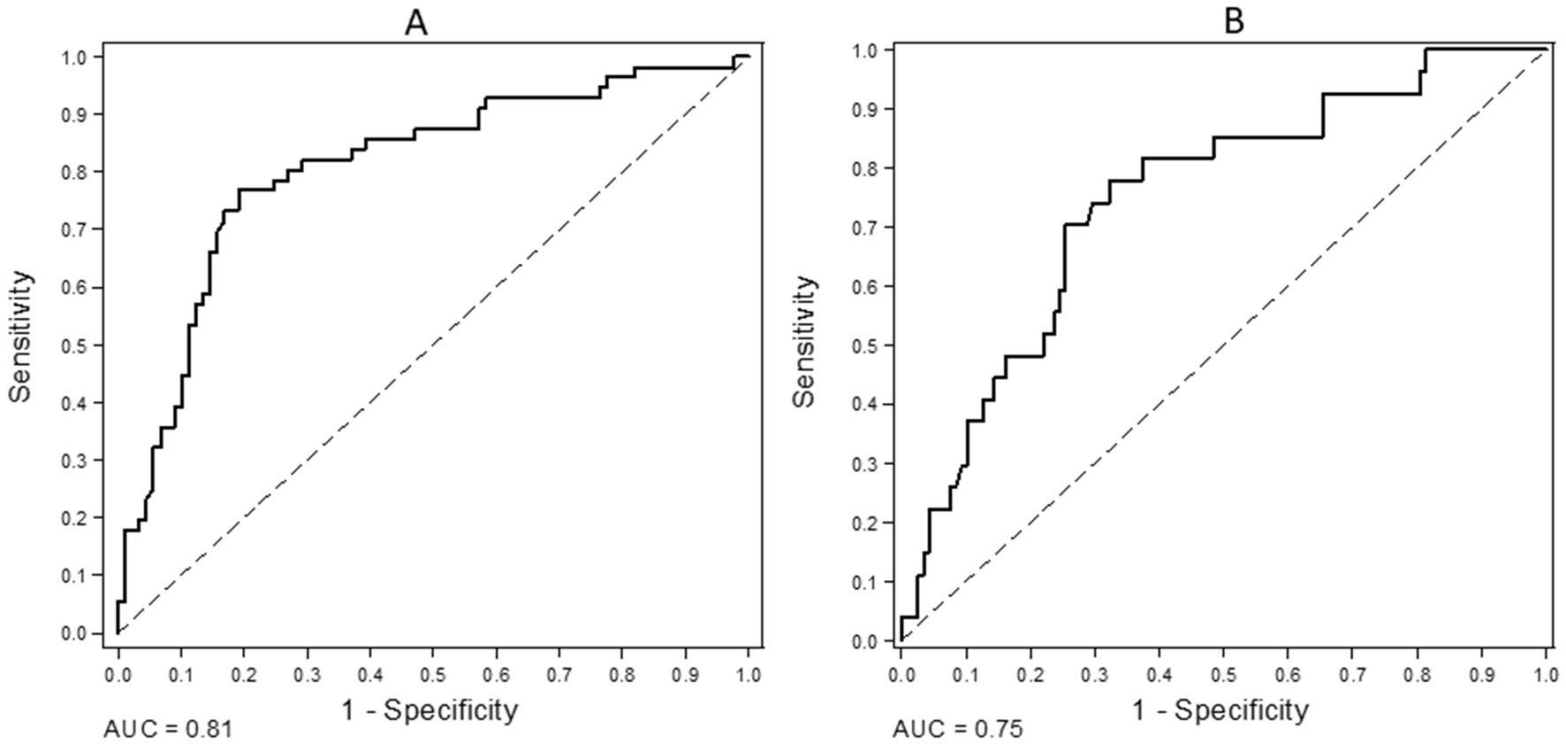
ROC curves for OPG—(**A**) All causes mortality (AUC 0.81, CI 95%: 0.73–0.88); (**B**) CV mortality (AUC: 0.75; CI 95%: 0.65–0.85).

The OPG cutoff value determined using the ROC curve for all causes mortality was 10 pmol/L (area under de curve [AUC] 0.81; CI 95%: 0.73–0.88; *p* < 0.001) and 10.08 pmol/L for CV mortality (AUC 0.75; CI 95%: 0.65–0.85, *p* < 0.001) (Fig. 1).

Clinical and biochemical characteristics of the 145 patients included in the study are summarized in Tables 1 and 2. The levels of OPG for the whole cohort had a median value of 8.91 (25–75th percentiles 6.51–12.43) pmol/L.

**Table 1:** Clinical and biochemical characteristics according to OPG levels.

	OPG ≤ 10.08 pmol/L (n = 85)	OPG > 10.08 pmol/L (n = 60)	*p* value*
Age (years)	55.0 ± 15.2	64.4 ± 13.0	< 0.001
Gender (male)	57 (67.1)	32 (53.3)	0.119
Diabetes (yes)	23 (27.1)	20 (33.3)	0.462
Dialysis (yes)	36 (42.4)	55 (91.7)	< 0.001
Hemodalysis (yes)	19 (22)	17 (28)	0.439
Peritoneal Dialysis (yes)	17 (20)	38 (63)	< 0.001
CVD	14 (16.5)	17 (28.3)	0.102
Hb (g/dL)	12.3 ± 2.0	11.1 ± 2.1	0.001
Albumin (g/dL)	4.2 ± 0.6	3.7 ± 0.6	0.001
P (mg/dL)	4.5 ± 1.7	4.8 ± 1.6	0.351
hsCRP (mg/dL)	5.5 ± 7.6	10.6 ± 14.7	0.017
IL-6 (pg/mL)	5.7 ± 11.9	10.4 ± 13.6	< 0.001
TNF-a (pg/mL)	16.9 ± 6.1	19.5 ± 9.3	0.063
PTX3 (pg/mL)	4.0 ± 2.4	6.1 ± 7	0.001
Fetuin (pg/mL)	0.43 ± 0.10	0.40 ± 0.11	0.109
FGF-23 (pg/mL)	4273 ± 11,106	5013 ± 12,023	0.019
S100A (ng/mL)	63.6 ± 74.4	101.8 ± 96.3	0.008
sRAGE (pg/mL)	1981 ± 1214	2577 ± 1124	< 0.001
Troponin I (ng/mL)	0.07 ± 0.07	0.19 ± 0.29	< 0.001
Diastolic dysfunction	52 (61.2)	47 (78.3)	0.031
Ejection fraction > 45%	76 (93.8)	50 (89.3)	0.356
LV mass index (g/m^2^)	58.1 ± 16.6	68.0 ± 23.9	0.008
IMT mean (cm)	0.67 ± 0.21	0.85 ± 0.42	0.001
Death (all-cause)	13 (15.3)	43 (71.7)	< 0.001
CV death	6 (7.1)	21 (35.0)	< 0.001

*Fisher exact test or Student’s t test; data of non-normal continuous variables were submitted to a logarithmic transformation; *p* < 0.05.

Quantitative variables are expressed as mean ± standard deviation. Categorical variables are expressed as frequencies (percent).

OPG, osteoprotegerin; Hb, hemoglobin; P, phosphate; hsCRP, high-sensitivity C-reactive protein; IL-6, interleukin-6; TNF-a, tumor necrosis factor alpha; PTX3, pentraxin-related protein; FGF-23, fibroblast growth factor 23; sRAGE, receptor for advanced glycation end products (RAGE) soluble form; LV, left ventricle; IMT, intima-media thickness; CV, cardiovascular.

**Table 2:** Clinical and biochemical characteristics according to CKD stage.

	CKD			p*
	Stage 3 (n = 26; 17.9%)	Stage 4 (n = 30; 20.7%)	Stage 5 (n = 89; 61.4%)	
Age (years)	61.8 ± 11	62.2 ± 14.3	57.0 ± 16.0	0.163
Gender (male)	16 (61.5)	21 (70)	52 (58.4)	0.530
Diabetes (yes)	7 (26.9)	13 (43.3)	23 (25.8)	0.182
OPG pmol/L	6.3 ± 2.2^a^	7.8 ± 3.2^a^	11.9 ± 5.8^b^	< 0.001
OPG > 10 pmol/L	2 (7.7)^a^	5 (16.7)^a^	53 (59.6)^b^	< 0.001
CVD	4 (15.4)	5 (16.7)	22 (24.7)	0.462
Hb (g/dL)	13.2 ± 1.7^a^	12.9 ± 2.1^a^	11.1 ± 1.9^b^	< 0.001
Albumin (g/dL)	4.4 ± 0.2	4.1 ± 0.9	3.8 ± 0.6	0.083
P (mg/dL)	3.4 ± 0.8^a^	3.7 ± 0.8^a^	5.2 ± 1.7^b^	< 0.001
hsCRP (mg/dL)	6.4 ± 10.9	7.2 ± 14.4	8.1 ± 10.4	0.099
IL-6 (pg/mL)	3.3 ± 4.7^a^	9.3 ± 18.6^b^	8.3 ± 11.8^b^	< 0.001
TNF-a (pg/mL)	14.8 ± 5.5^a^	15.6 ± 8.6^a^	19.6 ± 7.4^b^	0.003
PTX3 (pg/mL)	3.2 ± 1.6^a^	3.6 ± 2.1^a^	5.8 ± 3.7^b^	< 0.001
Fetuin (pg/mL)	0.43 ± 0.09	0.44 ± 0.07	0.41 ± 0.11	0.283
FGF-23 (pg/mL)	154 ± 103^a^	192 ± 196^a^	7386 ± 13,981^b^	< 0.001
S100A (ng/mL)	59.7 ± 74.1	52.9 ± 34.1	93.9 ± 97.6	0.166
sRAGE (pg/mL)	1539 ± 876^a^	1710 ± 1053^a^	2600 ± 1200^b^	< 0.001
Troponin I (ng/mL)	0.05 ± 0.04^a^	0.10 ± 0.11^a^	0.15 ± 0.25^b^	< 0.001
Diastolic dysfunction	21 (80.8)	24 (80)	54 (60.7)	0.050
Ejection fraction > 45%	2 (8.3)	2 (6.9)	7 (8.3)	0.969
LV mass index (g/m^2^)	62.5 ± 17.2	65.6 ± 25.6	60.6 ± 19.2	0.620
IMT mean (cm)	0.67 ± 0.22	0.72 ± 0.25	0.78 ± 0.37	0.379
Death (all-cause)	5 (19.2)^a^	4 (13.3)^a^	47 (52.8)^b^	< 0.001
CV death	1 (3.8)^a^	2 (6.7)^a^	24 (27.0)^b^	0.005

*Chi-square test or one-way ANOVA; data of non-normal continuous variables were submitted to a logarithmic transformation; *p* < 0.05.

^a,b^Different letters indicate significant difference (*p* < 0.05).

Quantitative variables are expressed as mean ± standard deviation. Categorical variables are expressed as frequencies (percent).

OPG, osteoprotegerin; Hb, hemoglobin; P, phosphate; hsCRP, high-sensitivity C-reactive protein; IL-6, interleukin-6; TNF-a, tumor necrosis factor alpha; PTX3, pentraxin-related protein; FGF-23, fibroblast growth factor 23; sRAGE, receptor for advanced glycation end products (RAGE) soluble form; LV, left ventricle; IMT, intima-media thickness; CV, cardiovascular.

Univariate correlations, assessed using Spearman’s rank, demonstrated that OPG levels were significantly associated with age (r = 0.37, *p* < 0.01), left ventricle mass index (r = 0.23, *p* = 0.007), S100 calcium-binding protein A12 (S100A12) (r = 0.21, *p* = 0.010), receptor for advanced glycation end products soluble form (sRAGE) (r = 0.33, *p* < 0.001), IL-6 (r = 0.39, *p* < 0.001), FGF-23 (r = 0.26, *p* = 0.001), hsCRP (r = 0.25, *p* = 0.003), troponin I (r = 0.54, *p* < 0.001), IMT (r = 0.40, *p* < 0.001), and ejection fraction, EF (r = −0.22, *p* = 0.010). However, when a multivariate analysis was performed, only age (*p* < 0.001), fibroblast growth factor-23 (*p* = 0.005), IL-6 (*p* = 0.017), troponin I (*p* = 0.002) and sRAGE (*p* = 0.002) were independently correlated with OPG levels.

### OPG levels and clinical outcome

Univariate analysis of mortality showed that elevated OPG levels (> 10 pg/mL for general causes death and > 10.08 pg/mL for CV death) were related to higher mortality for both general and cardiovascular causes (SHR 6.81; CI 95%: 3.69–12.6, *p* < 0.001 and SHR 5.47; CI 95%: 2.24–13.4, *p* < 0.001, respectively), together with levels of serum troponin (SHR = 7.73, CI 95%: 3.17–18.8, *p* < 0.001 and SHR 10.8, CI 95%: 4.79–22.8), *p* < 0.001, respectively) and log(sRAGE) levels (SHR = 1.47, CI 95%: 0.87–2.49, *p* = 0.150 and SHR 2.07; CI 95%: 1.07–3.95, *p* = 0.035, respectively), and treatment by dialysis (SHR 4.80, CI 95%: 2.17–10.6, *p* < 0.001 and SHR 8.73, CI 95%: 1.86–32.5, *p* = 0.003, respectively).

**Figure 2:**
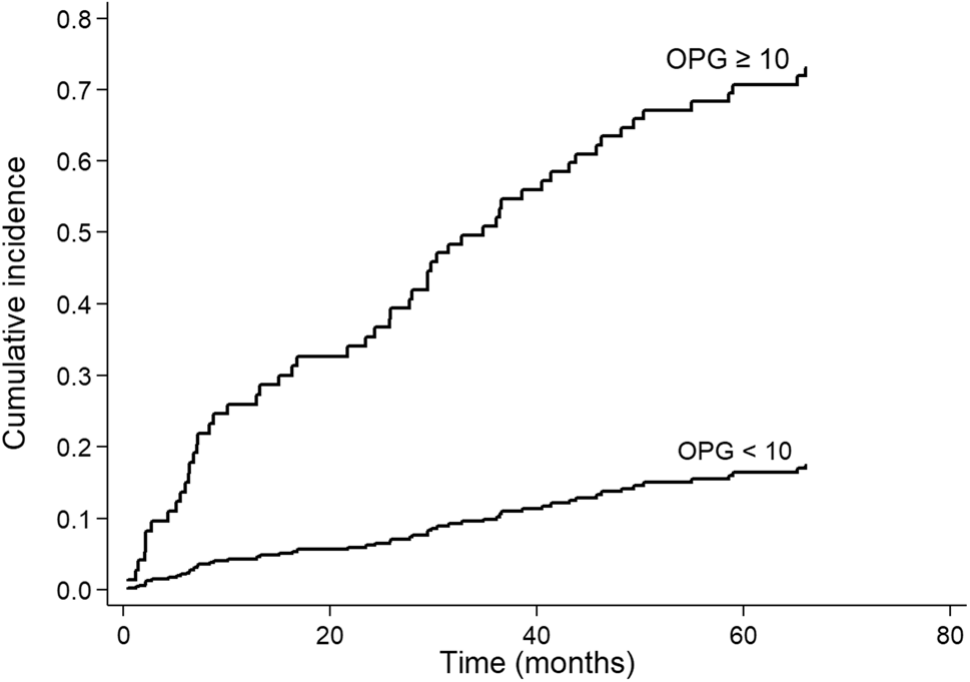
Aalen–Johansen cumulative incidence curves for all-cause mortality in 145 CKD patients with OPG levels ≤ 10 pmol/L or > 10 pmol/L.

**Figure 3:**
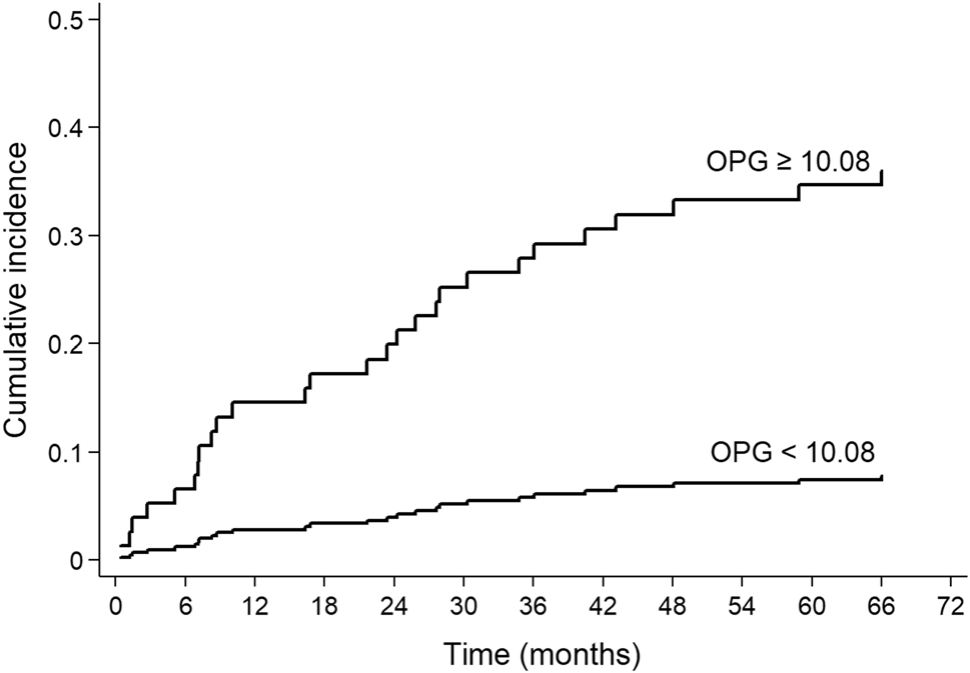
Aalen–Johansen cumulative incidence curves for cardiovascular death in 145 CKD patients with OPG levels ≤ 10.08 pmol/L or > 10.08 pmol/L.

Aalen–Johansen cumulative incidence curves showed a higher mortality for general (Fig. 2) and cardiovascular (Fig. 3) causes in the group with elevated OPG (> 10 pmol/L for general causes and > 10.08 pmol/L for CV causes) early in the follow-up period, with an increase in the gap over time (during the follow-up period, 25 patients had undergone kidney transplantation and 16 were lost to follow-up, of which 9 were in the non-dialysis group). The number of deaths was 56 (cardiovascular: 25; infection: 17; malignances: 4; and other causes: 10).

In multivariate analysis, in a model including gender and age, OPG was found to be an independent marker of all-cause and cardiovascular mortality. When presence of previous CVD and diabetes, and hsCRP, were added, the results were similar. However, when CKD stages were included in the analysis, OPG was an independent marker only for all-cause mortality. (Tables 3, 4).

**Table 3:** Multivariate analysis for all-cause mortality.

Model	Variable	*p* value^a^	SHR	CI 95%
Crude model	OPG	< 0.001	1.10	1.06–1.14
Model 1	OPG	< 0.001	1.08	1.05–1.12
	Age	0.079	1.02	1.00–1.05
	Sex (male)	0.145	0.68	0.40–1.14
Model 2	OPG	< 0.001	1.08	1.05–1.12
	Age	0.128	1.02	1.00–1.04
	Sex (male)	0.109	0.64	0.40–1.10
	Diabetes	0.654	0.87	0.48–1.58
	CVD	0.277	1.44	0.74–2.79
Model 3	OPG	< 0.001	1.08	1.04–1.12
	Age	0.121	1.02	1.00–1.05
	Sex (male)	0.089	0.62	0.36–1.08
	Diabetes	0.823	0.93	0.50–1.72
	CVD	0.371	1.40	0.68–2.85
	hsCRP	0.179	1.01	0.99–1.03
Model 4	OPG	0.048	1.04	1.00–1.08
	Age	0.005	1.04	1.01–1.07
	Sex (male)	0.389	0.78	0.45–1.37
	Diabetes	0.816	0.93	0.49–1.74
	CVD	0.643	1.18	0.59–2.36
	hsCRP	0.029	1.02	1.00–1.04
	**CKD stage**			
	3 (ref)^a^			
	4	0.164	0.35	0.08–1.53
	5	0.061	2.55	0.96–6.79

Fine and Gray model for mortality (any cause) considering transplant as competing risk.

^a^Stage 3 used as a reference for the analysis.

OPG, osteoprotegerin; CVD, cardiovascular disease; hsCRP, high-sensitivity C-reactive protein; CKD, Chronic Kidney Disease; SHR: subdistribution hazard ratio.

**Table 4:** Multivariate analysis for cardiovascular-related mortality.

Model	Variable	*p* value^a^	SHR	CI 95%
Crude model	OPG	< 0.001	1.10	1.05–1.15
Model 1	OPG	< 0.001	1.10	1.04–1.15
	Age	0.855	1.00	0.97–1.03
	Sex (male)	0.879	0.94	0.44–2.02
Model 2	OPG	< 0.001	1.10	1.04–1.16
	Age	0.929	1.00	0.97–1.03
	Sex (male)	0.833	0.92	0.43–1.99
	Diabetes	0.783	1.12	0.50–2.53
	CVD	0.426	1.43	0.60–3.42
Model 3	OPG	0.001	1.10	1.04–1.16
	Age	0.757	1.00	0.96–1.03
	Sex (male)	0.904	1.05	0.48–2.30
	Diabetes	0.687	1.18	0.52–2.68
	CVD	0.340	1.53	0.64–3.66
	hsCRP	0.885	1.00	0.96–1.04
Model 4	OPG	0.074	1.06	0.99–1.13
	Age	0.668	1.01	0.97–1.04
	Sex (male)	0.668	1.19	0.54–2.64
	Diabetes	0.616	1.24	0.54–2.83
	CVD	0.588	1.28	0.52–3.15
	hsCRP	0.869	1.00	0.96–1.04
	**CKD stage**			
	3 (ref)*			
	4	0.664	1.72	0.15–19.6
	5	0.105	5.34	0.71–40.4

Fine and Gray model for mortality (cardiovascular causes) considering transplant and other cause of mortality as competing risk.

^a^Stage 3 used as a reference for the analysis

OPG, osteoprotegerin; CVD, cardiovascular disease; hsCRP, high-sensitivity C-reactive protein; CKD, chronic kidney disease; SHR: subdistribution hazard ratio.

## Discussion

The main cause of death in our cohort was cardiovascular diseases, accounting for 45% (25 cases) of all deaths. This result is in line with those reported in the current literature, wherein large population studies have shown a progressive increase in the incidence of cardiovascular mortality with worsening renal function^17^.

The main finding in the present study was that OPG was a marker of higher cardiovascular mortality in patients with CKD, regardless of gender and age and other cardiovascular risk factors such as presence of diabetes, previous history of CVD as well as inflammation. We have been previously reported that an increased OPG concentration was associated with increased all-cause 3-year mortality risk among patients with CKD stages 3–5^13^. In the current study, the follow-up of this cohort was extended to 5, 5 years and, in addition to all-cause mortality risk, we also analyzed the association of OPG with cardiovascular mortality. Furthermore, the quality of the analysis was improved as we applied a ROC derived cut-off level of OPG in the survival analyses. The present results corroborate the results of several other studies, including those by Morena et al*.* and Matsubara et al*.* who found higher mortality in dialysis patients with high OPG levels^18, 19^. Recently, Alderson et al*.* reported that OPG levels were associated with increased mortality risk in the CKD population^8^. Kuźniewski et al*.* proposed that the relationship between OPG and tumor necrosis factor-related apoptosis-inducing ligand (TRAIL) would act as a marker of increased cardiovascular mortality risk in stage 5 CKD patients, and, in their study, isolated OPG values were also able to predict all-cause and cardiovascular mortality^20^. In a recent meta-analysis Huang et al. found a relationship between OPG and increased cardiovascular mortality in patients with CKD, however, cited as some limitations: most studies including only dialysis patients, lack of adjustment for potential confounders and the lack of several studies in the area^21^. Altogether these studies suggest that an elevated circulating concentration of OPG appears to be a marker for increased all-cause and cardiovascular mortality risk in patients with CKD.

Although OPG is classically associated with vascular calcification, the exact mechanisms by which OPG may lead to higher mortality are still unknown. One of the current theories is that OPG is also a marker of atherosclerotic disease and myocardial ischemia^22–26^. Our results demonstrated that circulating OPG levels were directly correlated with serum troponin levels, one of the main markers of myocardial damage, corroborating these data.

Regarding cardiovascular disease, another possible question would be the relationship between OPG and ventricular function, since it is possible that OPG can also be secreted by the heart^27^. While previous studies were divergent regarding this relationship, Lindberg et al*.* found a correlation between OPG levels and a lower ejection fraction in patients after an acute ischemic event in a study including 42 patients. However, Shetelig et al*.* did not find this relationship in 272 patients with coronary disease^28, 29^. While our study found no independent correlation between OPG and ejection fraction, diastolic dysfunction, or increased ventricular mass, OPG was a marker of increased mortality independent of the presence of previously known heart disease in patients with CKD, when this and other well established risk factors (age, sex, diabetes, and inflammation) were included in a multivariate analysis.

Some authors propose that high OPG levels may be a direct consequence of vascular inflammation^19^. Other studies have demonstrated that OPG expression can be induced by the vascular musculature through inflammatory cytokines, reinforcing this idea^30, 31^. However, our study did not find a correlation between inflammatory markers and OPG, and when an inflammation marker (hsCRP) was included in the multivariate analysis, OPG remained as an independent marker of cardiovascular mortality. Sigrist et al*.* reported similar results, in that high levels of OPG were associated with higher mortality independently of hsCRP levels in patients with CKD^32^.

Recently, Wieczorek-Surdacka et al.^33^ reported that elevated levels of OPG were associated with a worse renal and cardiovascular outcome in patients with chronic renal disease and stable coronary artery disease and that there was an independent relationship between elevated levels of OPG and low homoarginine (hArg)/symmetric dimethylarginine ratio (ADMA). However, our study did not include measurements of hArg or serum ADMA.

When CKD stages were included in the multivariate analysis, OPG was an independent marker of all-cause mortality but not cardiovascular mortality. This may to some extent reflect the link between elevated OPG levels and poor renal function^34^, and by the fact that cardiovascular mortality is increased in patients CKD stage 5, especially in those undergoing dialysis^35^.

## Conclusions

This study has some limitations that should be mentioned. First, this was an observational study, precluding any conclusions regarding causality. Second, the number of patients was relatively small, which did not allow us to include it in a specific multivariate analysis of mortality within non-dialysis group. Furthermore, only a single sample was collected, and the analysis was restricted to a certain time point, which may fail to reflect the natural course of the processes being studied. In addition, we did not measure other relevant markers that are associated with OPG and its actions such as RANKL and TRAIL or presence of vascular calcification asssed by coronary artery calcium score. Also, we do not have albuminuria measured in all cohort because most part of our dialysis patients did not have any residual function. Moreover, the lack of laboratory standardization to analyze albuminuria in a single urine sample made this analysis unfeasible. It would be necessary to collect 24 h urine samples which was not possible to perform in the present study.

In summary, we report that a high circulating OPG level was a prognostic indicator of increased all-cause and cardiovascular mortality risk in patients with CKD. These results suggest that OPG could be potentially useful in the prognostic evaluation of patients with CKD, as previously proposed by other authors. Future research should investigate correlations between OPG and other markers of cardiovascular mortality, such as the coronary artery calcium score, and explore whether therapies aimed at reducing the high circulating concentration of OPG in patients with CKD may reduce the high cardiovascular mortality in this patient population.

## Data Availability

The datasets used and/or analyzed during the current study are available from the corresponding author on reasonable request.
